# The role of artificial intelligence in surgical simulation

**DOI:** 10.3389/fmedt.2022.1076755

**Published:** 2022-12-14

**Authors:** Jay J. Park, Jakov Tiefenbach, Andreas K. Demetriades

**Affiliations:** ^1^ Department of General Surgery, Norfolk and Norwich University Hospital, Norwich, United Kingdom; ^2^ Edinburgh Medical School, University of Edinburgh, Edinburgh, United Kingdom; ^3^ Neurological Institute, Cleveland Clinic, Cleveland, OH, United States; ^4^ Department of Neurosurgery, Royal Infirmary of Edinburgh, Edinburgh, United Kingdom

**Keywords:** artificial intelligence (AI), surgical training, surgical simulation, machine learning, deep learning, virtual reality, augemented reality, mixed reality

## Abstract

Artificial Intelligence (AI) plays an integral role in enhancing the quality of surgical simulation, which is increasingly becoming a popular tool for enriching the training experience of a surgeon. This spans the spectrum from facilitating preoperative planning, to intraoperative visualisation and guidance, ultimately with the aim of improving patient safety. Although arguably still in its early stages of widespread clinical application, AI technology enables personal evaluation and provides personalised feedback in surgical training simulations. Several forms of surgical visualisation technologies currently in use for anatomical education and presurgical assessment rely on different AI algorithms. However, while it is promising to see clinical examples and technological reports attesting to the efficacy of AI-supported surgical simulators, barriers to wide-spread commercialisation of such devices and software remain complex and multifactorial. High implementation and production costs, scarcity of reports evidencing the superiority of such technology, and intrinsic technological limitations remain at the forefront. As AI technology is key to driving the future of surgical simulation, this paper will review the literature delineating its current state, challenges, and prospects. In addition, a consolidated list of FDA/CE approved AI-powered medical devices for surgical simulation is presented, in order to shed light on the existing gap between academic achievements and the universal commercialisation of AI-enabled simulators. We call for further clinical assessment of AI-supported surgical simulators to support novel regulatory body approved devices and usher surgery into a new era of surgical education.

## Introduction

In recent years, artificial intelligence (AI) technology has fuelled the advancement of surgical simulators, enhancing their accuracy and capabilities. Surgical simulators are increasingly used in modern medical practice to support preoperative planning and enhance the hands-on experience of training a surgeon. The application of modern AI technology can support simulators in providing personalised feedback to the user, while also automating an immersive surgical experience for visualisation of patient anatomy (1–5). In this review, we aim to discuss the current state of AI technology in surgical simulation. By reviewing the available literature and through our consolidated list of FDA/CE approved AI-enabled medical devices, we attempt to highlight recent advancements in the field as well as discuss some gaps between the research and industry communities. The key terms relevant to this article are summarised in Table 1.

**Table 1:** Definitions.

Terminology	Definition
Artificial intelligence (AI)	Mimicking of human intelligence through ability to predict, classify, predict, learn, plan, reason and/or perceive to make decisions
Machine learning (ML)	Sub-branch of AI technology that processes calculations and statistics to learn from data without human supervision
Deep learning (DL)	Subset of ML that uses neural networks to solve more complex challenges such as image, audio, and video classifications.
Artificial neural network (ANN)	A collection of simple interconnected algorithms that process information in response to external input
Convolutional neural network (CNN)	A deep learning neural network designed for processing structured arrays of data such as images.
Augmented reality (AR)	A class of ANN; deep learning neural network designed for processing structured arrays of data such as images.
Virtual reality (VR)	A computer-generated environment that replaces the real environment with a digital environment where users interact with virtual objects
Mixed reality (MR)	A hybrid environment that merges both real and virtual worlds with digital and physical objects that can interact in real-time
Extended reality (XR)	A complete immersive environment which integrates AR, VR, and MR technologies to provide an entire spectrum of reality to virtuality.
Head-mounted display (HMD)	A display device, worn on the head or as part of a helmet, that has a small display optic in front of one or each eye.

### The application of AI in surgical simulation assessment

AI can improve surgical training simulators by evaluating performance and providing personalised feedback to the end user (6). Several AI-based algorithms have been described in recent years. For instance, a neurosurgical group at McGill University, Montreal, developed a machine learning (ML) algorithm that classifies participants’ skill levels while performing a VR-based hemilaminectomy or brain tumour resection task (7, 8). More recently, the same group developed a “Virtual operative assistant (VOA)”—an open-sourced AI-based software that, in addition to determining the skill level, also provides personalised feedback in relation to expert proficiency performance benchmarks.

However, like any form of emerging technology, AI-driven performance evaluation and feedback generation remains imperfect with numerous limitations. Lam et al.'s systematic review explored several different ML techniques assessing surgical performance and noted the promising accuracy of ML-based assessment software, while delineating several major challenges and limitations of this technology (9). One limitation is that the surgical skill assessment of a simulated benchtop task might not accurately correlate with the trainee's performance in the operating room, as the real-life surgical environment is highly complex. Furthermore, there is a lack of validated assessment criteria against which participants’ performance should be assessed by the AI-algorithm. Another major barrier is the exorbitant volume of surgical data required to effectively train the ML-algorithms. Potential solutions to some of these challenges include developing a surgeon-led consensus statement outlining the core elements of a surgical technique to be evaluated by ML-algorithms and facilitating surgical data exchange through cross-institutional open-source initiatives (10).

Interestingly, we note that our review of the FDA/CE list did not identify any approved AI-powered software for appraising and providing feedback in surgical simulation. However, it is conceivable that such software may be commercially available but have not been evaluated through the regulatory process as they do not directly interact with patients (11).

### The application of AI in surgical visualisation

3D visualisation has been a major development in surgical simulation across all surgical specialties ranging from neurosurgery and orthopaedics to maxillofacial, plastics, and general surgery (12, 13). Such universal demand for 3D presurgical planning and its steadfast advancement since the 1980s testifies to the substantial benefits in reducing operation duration, blood loss, and hospital stay (14–18) whilst improving patient survival. Our review of the FDA/CE list identified 11 AI-enabled visualisation devices (Table 2). It is important to note that this list does not include visualisation technology without obvious AI application.

**Table 2:** List of FDA/CE approved AI-powered devices.

Device name	Approval date	FDA submission number	Type of approval	Other approval	Approval date	Description	Company	Country	Software function	Source
UNiD Spine Analyzer	5/24/2017	K170172	510 (k) premarket notification			Device that allows surgeons to view, measure images, and to perform generic spine related presurgical simulations for placement of surgical implants	Medicrea International (Device acquired by Medtronic)	USA	2D simulation	1,3
D2P	8/29/2019	K183489	510 (k) premarket notification	CE	10/2017	Pre-operative image segmentation and DICOM visualisation software for surgical planning	3D Systems, Inc.	USA	3D printing, VR simulation	1,2,3,4
HealthJOINT	12/04/2020	K202487	510 (k) premarket notification	NA	NA	Software for 3D reconstruction from 2D images for preoperative planning of knee orthopaedic surgical procedures	Zebra Medical Vision Ltd (Acquired by Nanox.ai)	USA	3D reconstruction	1,3
Innersight3D	NA	NA	NA	CE	06/2019	Modelling technology for surgery planning using models from MRI and CT scans	InnersightLabs Ltd.	UK	3D reconstruction	4
MEDIP PRO	11/07/2019	K191026	510 (k) premarket notification	CE	12/2019	Software interface and image segmentation system for transfer of DICOM imaging information from medical scanner to output file; pre-operative software for treatment planning	Medical IP Co., Ltd.	South Korea	3D reconstruction, 3D Printing, MR/XR Simulation	1,3,4,5
NemoScan	12/11/2019	K192571	510 (k) premarket notification	NA	NA	Diagnostic and treatment support for dental implantology	Software Nemotec S.L.	Spain	3D reconstruction, 3D Printing	1,4
Profuse CAD	11/21/2018	K173744	510 (k) premarket notification	NA	NA	Standalone Computer Aided Detection (CAD) software used to visualise, analyse, plan and interpret CT, MRI, PET images	Eigen	USA	3D reconstruction	2
SurgicalAR	5/13/2019	K190764	510 (k) premarket notification	NA	NA	Display of medical images and other healthcare data, including functions for image review, basic measurements and 3D visualization	Medivis, Inc.	USA	MR/XR Simulation	1,4
Synapse 3D, Synapse 3D Base Tools v6.1	02/09/2021	K203103	510 (k) premarket notification	NA	NA	Software for advanced processing and analysis of imaging aiding in surgical decision support and preoperative simulation	Fujifilm Corporation	USA	3D Reconstruction	1
Syngo Application Software	06/09/2017	K170747	510 (k) premarket notification	CE	03/2017	Provides real-time viewing, image manipulation, 3D-visualisation, communication, and storage of medical images	Siemens Medical Solutions, USA, Inc.	USA	3D reconstruction	1,2,4
VSI Holomedicine	NA	NA	NA	CE	12/2019	Surgery support through mixed reality	apoQlar GmbH	Germany	VR simulation, MR/XR Simulation	4

1. Artificial Intelligence and Machine Learning (AI/ML) Medical Devices [Internet]. U.S. Food and Drug Administration. 2021. Available from: https://www.fda.gov/medical-devices/software-medical-device-samd/artificial-intelligence-and-machine-learning-aiml-enabled-medical-devices#resources.

2. Wu E, Wu K, Daneshjou R, Ouyang D, Ho D, Zou J. How medical AI devices are evaluated: limitations and recommendations from an analysis of FDA approvals. Nature Medicine. 2021;27(4):582-584.

3. Dreyer K, Wald C, Allen Jr. B, Agarwal S, Bizzo B, Gichoya J et al. AI Central [Internet]. AI Central. 2022. Available from: https://aicentral.acrdsi.org/.

4. Muehlematter U, Daniore P, Vokinger K. Approval of artificial intelligence and machine learning-based medical devices in the USA and Europe (2015–20): a comparative analysis. The Lancet Digital Health. 2021;3(3):e195-e203.

5. STAT Database: AI Tools [Internet]. Statnews.com. 2021. Available from: https://www.statnews.com/wp-content/uploads/2021/02/STAT_FDA_cleared-_AI_tools.xlsx.

**Figure 1:**
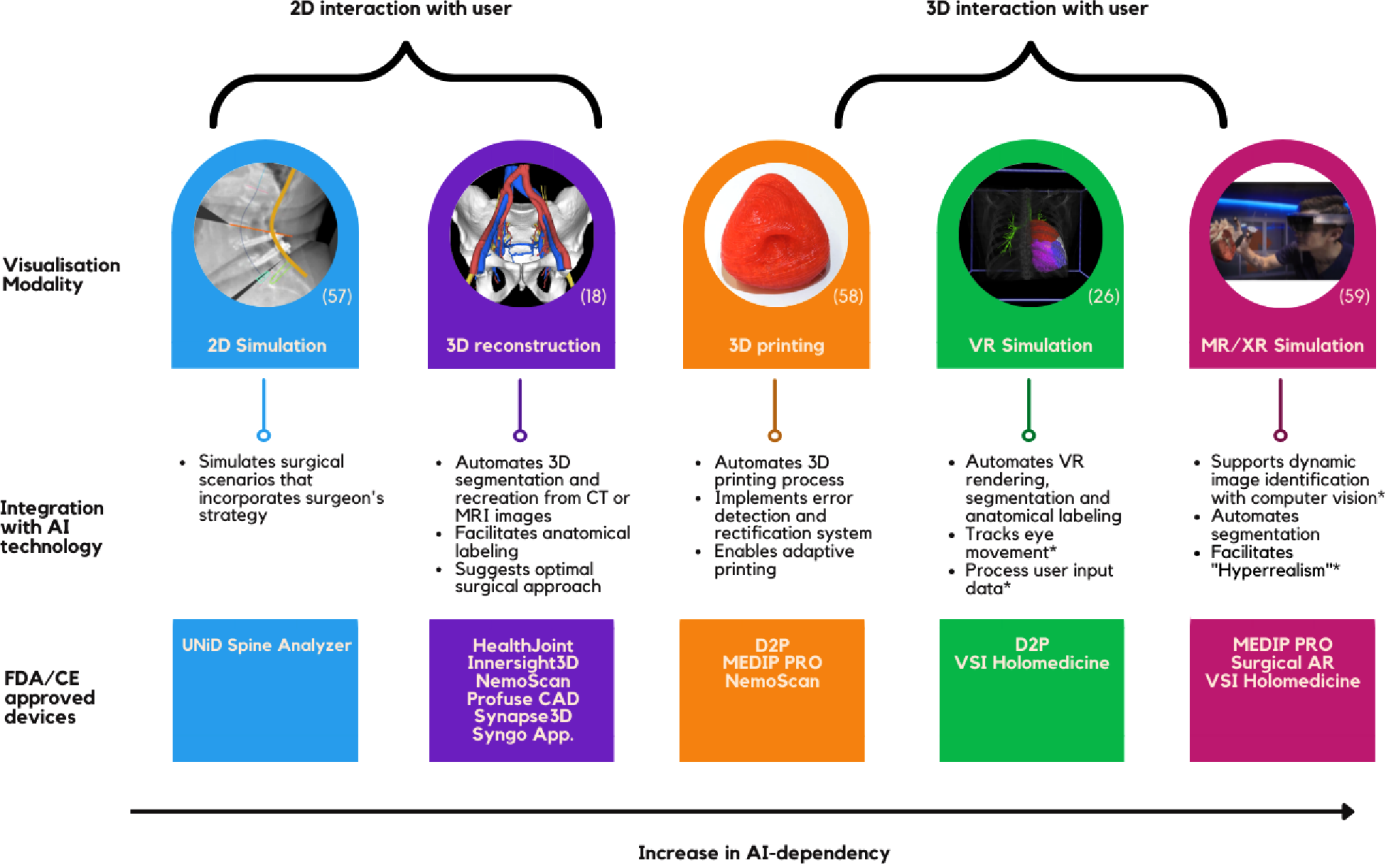
Summary of AI function in visualisation technology.

The most widely accepted technique for creating 3D simulations from 2D images involves convoluted neural network (CNN) algorithms. AI can complement this method by facilitating 3D segmentation and anatomical labeling (19–21). In addition, AI plays a crucial role in facilitating advanced visualisation modalities such as 3D printing, virtual reality (VR) simulation, and extended reality (XR) environment (Figure 1).

3D-printed simulators have been shown to facilitate preoperative planning, improve surgical outcomes and decrease operation duration (22); however, several challenges have hindered its daily application (23). AI has removed the largest barrier of implementing 3D printing—the need for expert knowledge in the 3D-printing process and computer-based design. Manual processes have been automated through AI algorithms that can efficiently process large amounts of data needed to prefabricate a model, convert through slicer, detect, and rectify any errors, and print. Moreover, AI-driven fabrication that adopts real-time adaptation poses a solution to 3D-printing's challenge of reproducing in-vivo tissue (24). AI technology is essential to support a VR environment and automating the workflow. Software such as DICOM to Print (D2P) and MEDIP PRO produces instantaneous 3D Printing and VR rendering options. Moreover, AI is effectively used for segmentation and anatomical annotation of the VR environment and, more importantly, processing user input needed to facilitate user interaction (25, 26). Furthermore, AI is required to support the XR-environment—the most practical form and the holy-grail of surgical simulation. In conjunction with computer vision technology, deep learning (DL) based object detection allows dynamic image identification (27). Such complex feedback processing from DL models bridges the mismatch between virtual and the real world in 3D holographic projections (28).

The simulation techniques are also integral in surgical training. VSI Holomedicine, for example, provides a platform for both preoperative planning and anatomical education. Furthermore, hyperrealism is an AR concept used in training where artificial objects, such as a silicon replica, are visually enhanced by DL algorithms to simulate a realistic scene of surgery (29).

## Discussion

### Technological challenges

Current AI frameworks used in surgical simulation have some technological limitations. To begin with, AI-powered feedback platforms could address the challenges experienced with the VOA (3). For example, VOA is restricted to categorising two expertise groups, whilst machine learning techniques such as artificial neural networks can be utilised to classify multiple groups of expertise (9, 30). VOA also highlighted the downfalls of using linear machine algorithms; there were instances of misclassification due to high positive scoring metrics overcompensating for other negative scoring metrics (3). Moreover, recent clinical experience with AI models demonstrated difficulties in measuring subjective combinations of actions such as “instrument handling (31).” In addition to overcoming these fundamental obstacles, a leap to translation will require succeeding platforms to be able to recognise and capture different frames from an entire simulation, in order to account for users’ variety of approaches and techniques that can adequately fulfil a single metric (3).

As seen in Figure 1, segmentation and anatomical labelling seems to be the major application of AI in surgical simulation; however, generalisability of these algorithms remains a challenge (32). Although segmentation has proven its efficacy in small clinical studies, its accuracy is yet to reach perfection (33). Studies reported that their algorithms failed to identify certain structures such as complex vasculature or certain types of tumours and that their accuracy can be highly dependent on the fidelity of different imaging modalities (32–34). Furthermore, to train AI algorithms to an absolute precision without bias and to be generalisable, experts are needed to construct comprehensive datasets which require numerous annotations and segmentation of anatomical structures. However, this manual process is tedious and resource intensive, hence, this approach cannot be translated into clinical practice (35). Such technical impediments are closely related to the fact that AI segmentation and anatomical labelling technology has varying progress across different specialties, pathologies, and procedures. Technologies currently in practice such as Syngo Application Software allow automated segmentation across several different specialties. Nevertheless, there is a lack of an overarching AI framework that can learn, train, and segment or label any anatomical structures in a human body. Some intraoperative AI models have been applying deep learning architectures to predict objects that were previously unseen by the AI (36). Albeit its infancy, this seems to be the future in AI-powered segmentation and anatomical labelling.

Additional technical challenges in the visualisation field are attributed to the intrinsic limitations of technological devices, [i.e., VR-sickness, head-mounted-displays (HMD), computing power of graphic processors] which is beyond the scope of this review. However, few AI-based solutions are proposed to overcome these challenges. VR-sickness is commonly experienced due to the delay between sensory input and VR. To reduce such latency, CNN models are employed to improve gaze-tracking by pre-rendering subsequent scenes and predicting future frames (37, 38). Another ground breaking area of development is in producing 3D displays for human eyes. Current AR/VR displays display 2D images to each of users’ eyes instead of 3D which is how individuals perceive the real world. Choi et al. recently proposed an AI algorithm and calibration technique for producing 3D images, namely the “Neural holography,” that allows a more realistic fidelity of VR rendering, comparable to that of an LCD display (39).

### Affordable cost

At the moment, the use of AI-based simulation in healthcare comes at a significant financial cost. As traditional practice for both surgical training and pre-operative planning rely on largely inexpensive methods such as near-peer teaching and group discussions, the introduction of AI-based simulators incurs additional costs. The price of such simulators is rarely publicly disclosed and varies greatly depending on quality, capabilities, and use cases. For instance, none of the companies we identified in our FDA/CE database search provided public information on the prices of their product. However, it is well known that the cost of simulator technology is steep. According to some estimates, the price to equip a simulation lab can range anywhere between $100,000 to several millions (40). The use of AI-algorithms adds an additional layer of costs, especially when considering that such algorithms need to be regularly updated to ensure optimal performance and reliability (41).

It is, therefore, important to understand if the clinical benefit from AI-driven surgical simulators would justify the significant costs associated with their purchase and maintenance. Interestingly, the majority of published research failed to yet report a significant superiority of surgical simulators as compared to traditional methods in the realm of surgical education (42). For instance, Madan et al. reported that substituting virtual reality trainers with an inanimate box does not decrease the rate and level of laparoscopic skill acquisitions. Similarly, a systematic review by Higgins et al. failed to identify any studies directly comparing the surgical simulation with traditional surgical skill development in the operating room (43). However, in the area of surgical visualisation for pre-operative planning, there is some, albeit still limited, evidence that supports the superiority of AI-driven simulators compared to traditional surgical techniques (14, 15, 18).

At the same time, it is important to explore if the cost of AI-driven surgical simulators can be reduced while maintaining the value they offer in patient care. The concept of “fidelity” refers to how well a surgical simulator represents reality (44). A range of studies found that higher fidelity simulators were rarely superior to their lower fidelity counterparts in the area of surgical education (45, 46). However, perceptual fidelity might prove to be important in simulators aiming to support surgical visualisation and preoperative planning process (47), although there is currently limited literature on the topic. Therefore, the choice to pursue low-cost, low-fidelity simulator variants needs to be carefully reviewed and balanced with the extent of benefits they can provide.

In addition to pursuing lower-fidelity simulators, it is reasonable to expect the price of simulators will be kept within a reasonable range with growing market competition and widespread adoption of this technology. Furthermore, national healthcare services and large healthcare institutions may have the negotiating power to further reduce the prices when supplying their hospitals with AI-driven surgical simulators and related equipment.

### Regulatory challenges

Presently, a clear overview of all approved AI-based medical devices and algorithms does not exist (48). Few efforts are underway to curate an inclusive database of these devices, in which FDA is at the forefront (49–51). Additionally, multiple other regulatory bodies such as the UK's Medicines and Healthcare products Regulatory Agency (MHRA), are making headway in developing regulatory guidelines to ensure safety and effectiveness of AI-powered devices and softwares (52). As valuable as these national endeavours are, it is crucial for internationally governing bodies to bring forth a guideline for implementing AI-powered surgical simulators universally (41). International Medical Device Regulators Forum (IMDRF) is a prime example of a group that can strive towards this goal (53).

To our knowledge, there are only a handful of AI-powered surgical simulators that are regulatory approved. Nevertheless, we emphasise the need for strict scrutiny of these products to ensure patient safety, transparency, and cyber security (41, 54). Although feedback softwares do not directly interact with patients, quality of training indirectly impacts patient care. For AI-based surgical simulation assessments to become the norm, like flight simulators, products should be driven to adhere to rigid requirements that uplift the quality of surgical training. From our experience with reviewing approved preoperative planning simulators, we highlight the significance of regulatory approval for several reasons. Products are often exaggerated or falsely marketed to be AI-powered, to boost their sales (48). Additionally, even for approved devices, the extent of use or the function of AI in their devices are only vaguely communicated to the public and the users. Ultimately, as preoperative simulators hold and analyse important patient information, they need to strictly abide by established data security guidelines for medical devices. Some preoperative simulators aim to automate the decision-making process which makes regulation even more pivotal. Despite the lack of data protection guidelines for AI specifically, the General Data Protection Regulation (GDPR) for example, contains elements that are relevant to such AI-based surgical planning simulators, including use of personal data, profiling, and automation of decision making (55). FDA's most recent endeavours share similar notions (50, 51).

Moving forward, global organisations like IMDRF need to corroborate on a consensus on AI regulation since the aforementioned issues are ubiquitous around the globe. As we aim towards universalisation of AI-based surgical simulators, legal and ethical issues regarding accountability and performance transparency of AI-based surgical simulators are crucial components that should be overseen by such governing bodies (54, 56).

## Conclusion

**Figure 2:**
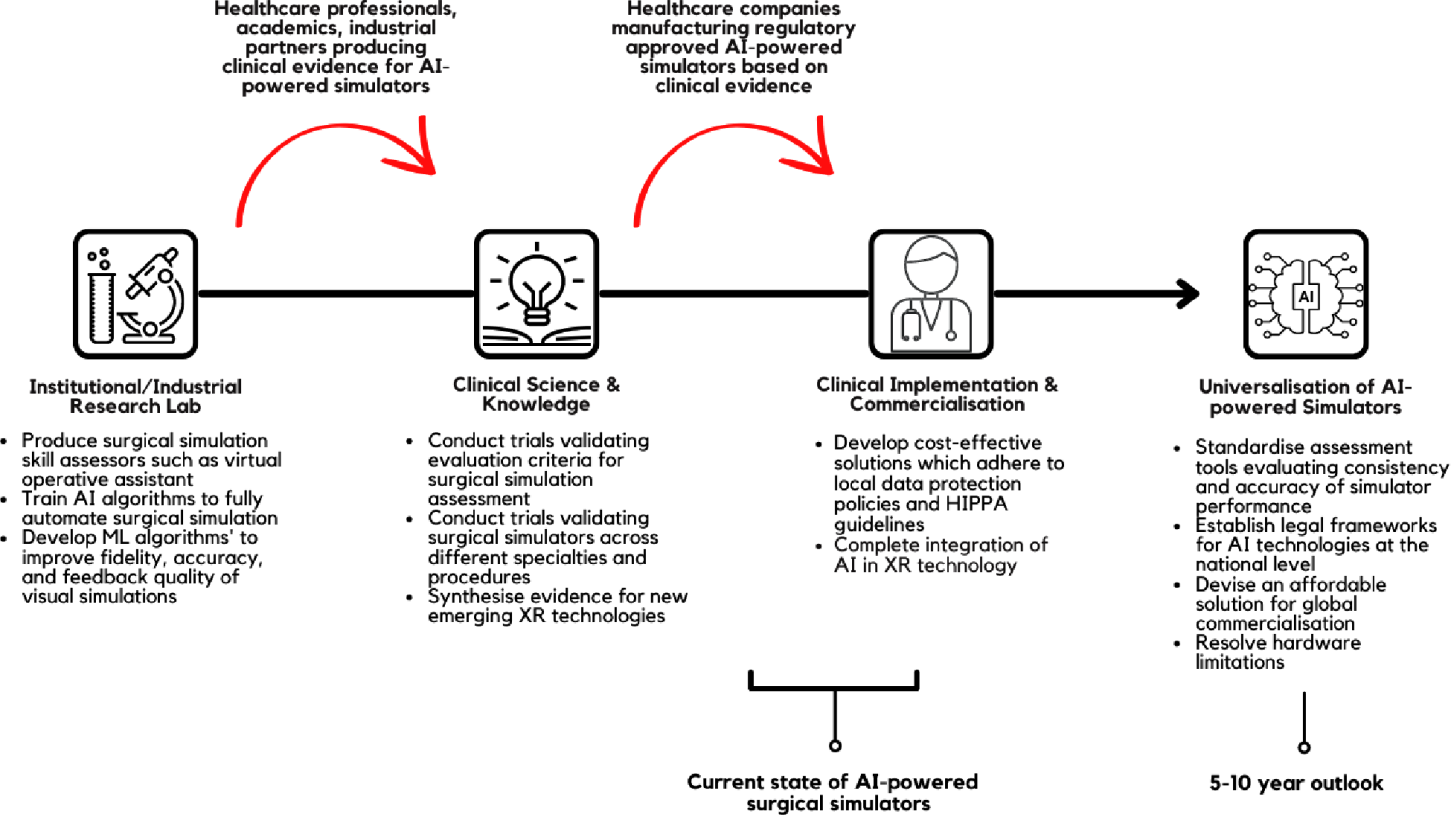
Recommendation and suggestions for AI-powered surgical simulators.

Application of AI has the potential to enhance the quality of surgical simulators and expand their capabilities. In this review article, we have discussed the elements of surgical simulation which are enhanced using AI-driven technologies and reviewed the available literature and FDA/CE-approved products. We summarised the current landscape of AI in surgical simulations and provided suggestions for further clinical implementation in Figure 2. It is promising to see clinical evidence and technological reports attesting to the efficacy of AI-supported surgical simulators. However, the barriers to wide-spread commercialisation of these devices and software are complex and multifactorial. High implementation and production cost, scarcity of reports evidencing the superiority of such technology, and intrinsic technological limitations remain at the forefront. With this in mind, we call for further clinical assessment of AI-supported surgical simulators to support novel regulatory body approved devices and usher surgery into a new era of surgical education.

## Data Availability

The original contributions presented in the study are included in the article/Supplementary Material, further inquiries can be directed to the corresponding author.

## References

[1] Bakshi SK Lin SR Ting DSW Chiang MF Chodosh J. The era of artificial intelligence and virtual reality: transforming surgical education in ophthalmology. Br J Ophthalmol. (2021) 105(10):1325–8. 10.1136/bjophthalmol-2020-316845 32816750

[2] Bravo J Wali AR Hirshman BR Gopesh T Steinberg JA Yan B Robotics and artificial intelligence in endovascular neurosurgery. Cureus. (2022) 14(3):e23662. 10.7759/cureus.23662 35371874 PMC8971092

[3] Mirchi N Bissonnette V Yilmaz R Ledwos N Winkler-Schwartz A Del Maestro RF. The virtual operative assistant: an explainable artificial intelligence tool for simulation-based training in surgery and medicine. PLoS One. (2020) 15(2):e0229596. 10.1371/journal.pone.0229596 32106247 PMC7046231

[4] Winkler-Schwartz A Bissonnette V Mirchi N Ponnudurai N Yilmaz R Ledwos N Artificial intelligence in medical education: best practices using machine learning to assess surgical expertise in virtual reality simulation. J Surg Educ. (2019) 76(6):1681–90. 10.1016/j.jsurg.2019.05.015 31202633

[5] Winkler-Schwartz A Yilmaz R Mirchi N Bissonnette V Ledwos N Siyar S Machine learning identification of surgical and operative factors associated with surgical expertise in virtual reality simulation. JAMA Network Open. (2019) 2(8):e198363. 10.1001/jamanetworkopen.2019.8363 31373651

[6] Sewell C Morris D Blevins NH Dutta S Agrawal S Barbagli F Providing metrics and performance feedback in a surgical simulator. Comput Aided Surg. (2008) 13(2):63–81. 10.3109/10929080801957712 18317956

[7] Siyar S Azarnoush H Rashidi S Del Maestro RF. Tremor assessment during virtual reality brain tumor resection. J Surg Educ. (2020) 77(3):643–51. 10.1016/j.jsurg.2019.11.011 31822389

[8] Bissonnette V Mirchi N Ledwos N Alsidieri G Winkler-Schwartz A Del Maestro RF. Artificial intelligence distinguishes surgical training levels in a virtual reality spinal task. J Bone Joint Surg Am. (2019) 101(23):e127. 10.2106/JBJS.18.01197 31800431 PMC7406145

[9] Lam K Chen J Wang Z Iqbal FM Darzi A Lo B Machine learning for technical skill assessment in surgery: a systematic review. NPJ Digit Med. (2022) 5(1):24. 10.1038/s41746-022-00566-0 35241760 PMC8894462

[10] Maier-Hein L Eisenmann M Sarikaya D März K Collins T Malpani A Surgical data science - from concepts toward clinical translation. Med Image Anal. (2022) 76:102306. 10.1016/j.media.2021.102306 34879287 PMC9135051

[11] Administration USFD. FDA rules and regulations (2020). Available at: https://www.fda.gov/regulatory-information/fda-rules-and-regulations

[12] Soon DSC Chae MP Pilgrim CHC Rozen WM Spychal RT Hunter-Smith DJ. 3D Haptic modelling for preoperative planning of hepatic resection: a systematic review. Ann Med Surg. (2016) 10:1–7. 10.1016/j.amsu.2016.07.002 PMC4959920 27489617

[13] Boaro A Moscolo F Feletti A Polizzi GMV Nunes S Siddi F Visualization, navigation, augmentation. The ever-changing perspective of the neurosurgeon. Brain Spine. (2022) 2:100926. 10.1016/j.bas.2022.100926 36248169 PMC9560703

[14] Fang CH Tao HS Yang J Fang ZS Cai W Liu J Impact of three-dimensional reconstruction technique in the operation planning of centrally located hepatocellular carcinoma. J Am Coll Surg. (2015) 220(1):28–37. 10.1016/j.jamcollsurg.2014.09.023 25456781

[15] Fang CH Liu J Fan YF Yang J Xiang N Zeng N. Outcomes of hepatectomy for hepatolithiasis based on 3-dimensional reconstruction technique. J Am Coll Surg. (2013) 217(2):280–8. 10.1016/j.jamcollsurg.2013.03.017 23870220

[16] Uchida M. Recent advances in 3D computed tomography techniques for simulation and navigation in hepatobiliary pancreatic surgery. J Hepatobiliary Pancreat Sci. (2014) 21(4):239–45. 10.1002/jhbp.82 24464989

[17] Mutter D Dallemagne B Bailey C Soler L Marescaux J. 3D Virtual reality and selective vascular control for laparoscopic left hepatic lobectomy. Surg Endosc. (2009) 23(2):432–5. 10.1007/s00464-008-9931-y 18443871

[18] Miyamoto R Oshiro Y Nakayama K Kohno K Hashimoto S Fukunaga K Three-dimensional simulation of pancreatic surgery showing the size and location of the main pancreatic duct. Surg Today. (2017) 47(3):357–64. 10.1007/s00595-016-1377-6 27368278

[19] Hamabe A Ishii M Kamoda R Sasuga S Okuya K Okita K Artificial intelligence-based technology to make a three-dimensional pelvic model for preoperative simulation of rectal cancer surgery using MRI. Ann Gastroenterol Surg. (2022) 6(6):788–94. 10.1002/ags3.12574 36338585 PMC9628238

[20] Kim H Jung J Kim J Cho B Kwak J Jang JY Abdominal multi-organ auto-segmentation using 3D-patch-based deep convolutional neural network. Sci Rep. (2020) 10(1):6204. 10.1038/s41598-020-63285-0 32277135 PMC7148331

[21] Neves CA Tran ED Kessler IM Blevins NH. Fully automated preoperative segmentation of temporal bone structures from clinical CT scans. Sci Rep. (2021) 11(1):116. 10.1038/s41598-020-80619-0 33420386 PMC7794235

[22] Tack P Victor J Gemmel P Annemans L. 3D-printing techniques in a medical setting: a systematic literature review. Biomed Eng Online. (2016) 15(1):115. 10.1186/s12938-016-0236-4 27769304 PMC5073919

[23] Segaran N Saini G Mayer JL Naidu S Patel I Alzubaidi S Application of 3D printing in preoperative planning. J Clin Med. (2021) 10(5):1–13. 10.3390/jcm10050917 PMC7956651 33652844

[24] Zhu Z Ng DWH Park HS McAlpine MC. 3D-printed multifunctional materials enabled by artificial-intelligence-assisted fabrication technologies. Nat Rev Mater. (2021) 6(1):27–47. 10.1038/s41578-020-00235-2

[25] Gupta D Hassanien AE Khanna A. Advanced computational intelligence techniques for virtual reality in healthcare. 1 ed. Switzerland, AG: Springer Cham (2020).

[26] Sadeghi AH Maat A Taverne Y Cornelissen R Dingemans AC Bogers A Virtual reality and artificial intelligence for 3-dimensional planning of lung segmentectomies. JTCVS Tech. (2021) 7:309–21. 10.1016/j.xjtc.2021.03.016 34318279 PMC8312141

[27] Devagiri JS Paheding S Niyaz Q Yang X Smith S. Augmented reality and artificial intelligence in industry: trends, tools, and future challenges. Expert Syst Appl. (2022) 207:118002. 10.1016/j.eswa.2022.118002

[28] Morimoto T Kobayashi T Hirata H Otani K Sugimoto M Tsukamoto M XR (extended reality: virtual reality, augmented reality, mixed reality) technology in spine medicine: status quo and quo vadis. J Clin Med. (2022) 11(2):470. 10.3390/jcm11020470 PMC8779726 35054164

[29] Wang DD Qian Z Vukicevic M Engelhardt S Kheradvar A Zhang C 3D Printing, computational modeling, and artificial intelligence for structural heart disease. JACC Cardiovascular Imaging. (2021) 14(1):41–60. 10.1016/j.jcmg.2019.12.022 32861647

[30] Reich A Mirchi N Yilmaz R Ledwos N Bissonnette V Tran DH Artificial neural network approach to competency-based training using a virtual reality neurosurgical simulation. Oper Neurosurg. (2022) 23(1):31–9. 10.1227/ons.0000000000000173 35726927

[31] Fazlollahi AM Bakhaidar M Alsayegh A Yilmaz R Winkler-Schwartz A Mirchi N Effect of artificial intelligence tutoring vs expert instruction on learning simulated surgical skills among medical students: a randomized clinical trial. JAMA Network Open. (2022) 5(2):e2149008. 10.1001/jamanetworkopen.2021.49008 35191972 PMC8864513

[32] Hamabe A Ishii M Kamoda R Sasuga S Okuya K Okita K Artificial intelligence–based technology for semi-automated segmentation of rectal cancer using high-resolution MRI. PLoS One. (2022) 17(6):e0269931. 10.1371/journal.pone.0269931 35714069 PMC9205476

[33] Ding X Zhang B Li W Huo J Liu S Wu T Value of preoperative three-dimensional planning software (AI-HIP) in primary total hip arthroplasty: a retrospective study. J Int Med Res. (2021) 49(11):1–21. 10.1177/03000605211058874. PMC8597071 34775845

[34] Chidambaram S Stifano V Demetres M Teyssandier M Palumbo MC Redaelli A Applications of augmented reality in the neurosurgical operating room: a systematic review of the literature. J Clin Neurosci. (2021) 91:43–61. 10.1016/j.jocn.2021.06.032 34373059

[35] Chen Z Zhang Y Yan Z Dong J Cai W Ma Y Artificial intelligence assisted display in thoracic surgery: development and possibilities. J Thorac Dis. (2021) 13(12):6994–7005. 10.21037/jtd-21-1240 35070382 PMC8743398

[36] Nakanuma H Endo Y Fujinaga A Kawamura M Kawasaki T Masuda T An intraoperative artificial intelligence system identifying anatomical landmarks for laparoscopic cholecystectomy: a prospective clinical feasibility trial (J-SUMMIT-C-01). Surg Endosc. (2022). 10.1007/s00464-022-09678-w 36261644

[37] Zhang M Ma KT Lim JH Zhao Q Feng J. Deep future gaze: gaze anticipation on egocentric videos using adversarial networks. 2017 IEEE conference on computer vision and pattern recognition (CVPR); 2017 July 21-26 (2017).

[38] Xu Y Dong Y Wu J Sun Z Shi Z Yu J Gaze prediction in dynamic 360° immersive videos. IEEE Conference on computer vision and pattern recognition (CVPR) (2018).

[39] Choi S Gopakumar M Peng Y Kim J Wetzstein G. Neural 3D holography: learning accurate wave propagation models for 3D holographic virtual and augmented reality displays. ACM Trans Graph. (2021) 40(6):240. 10.1145/3478513.3480542

[40] Bernier GV Sanchez JE. Surgical simulation: the value of individualization. Surg Endosc. (2016) 30(8):3191–7. 10.1007/s00464-016-5021-8 27338581

[41] He J Baxter SL Xu J Xu J Zhou X Zhang K. The practical implementation of artificial intelligence technologies in medicine. Nat Med. (2019) 25(1):30–6. 10.1038/s41591-018-0307-0 30617336 PMC6995276

[42] Madan AK Frantzides CT. Substituting virtual reality trainers for inanimate box trainers does not decrease laparoscopic skills acquisition. J Soc Laparoendosc Surg. (2007) 11(1):87–9. PMC3015783 17651563

[43] Higgins M Madan C Patel R. Development and decay of procedural skills in surgery: a systematic review of the effectiveness of simulation-based medical education interventions. Surgeon. (2021) 19(4):e67–77. 10.1016/j.surge.2020.07.013 32868158

[44] Lewis R Strachan A Smith MM. Is high fidelity simulation the most effective method for the development of non-technical skills in nursing? A review of the current evidence. Open Nurs J. (2012) 6:82–9. 10.2174/1874434601206010082 22893783 PMC3415625

[45] Norman G Dore K Grierson L. The minimal relationship between simulation fidelity and transfer of learning. Med Educ. (2012) 46(7):636–47. 10.1111/j.1365-2923.2012.04243.x 22616789

[46] Grober ED Hamstra SJ Wanzel KR Reznick RK Matsumoto ED Sidhu RS The educational impact of bench model fidelity on the acquisition of technical skill: the use of clinically relevant outcome measures. Ann Surg. (2004) 240(2):374–81. 10.1097/01.sla.0000133346.07434.30 15273564 PMC1356416

[47] Stoyanov D Mylonas GP Lerotic M Chung AJ Yang GZ. Intra-operative visualizations: perceptual fidelity and human factors. J Disp Technol. (2008) 4(4):491–501. 10.1109/JDT.2008.926497

[48] Benjamens S Dhunnoo P Meskó B. The state of artificial intelligence-based FDA-approved medical devices and algorithms: an online database. NPJ Digit Med. (2020) 3:118. 10.1038/s41746-020-00324-0 32984550 PMC7486909

[49] U.S Food & Drug Administration. Artificial intelligence and machine learning (AI/ML)-enabled medical devices (2022). Available at: https://www.fda.gov/medical-devices/software-medical-device-samd/artificial-intelligence-and-machine-learning-aiml-enabled-medical-devices

[50] U.S Food & Drug Administration. Artificial intelligence/machine learning (AI/ML)-based software as a medical device (SaMD) action Plan (2021).

[51] U.S Food & Drug Administration. Proposed regulatory framework for modifications to artificial intelligence/machine learning (AI/ML)-based software as a medical device (SaMD). Department of Health and Human Services (United States) (2019).

[52] Clarke O. UK digital health – the future of software as a medical device (2021).

[53] International Medical Device Regulators Forum. Artificial intelligence medical devices (2022). Available at: https://www.imdrf.org/working-groups/artificial-intelligence-medical-devices.

[54] Kiseleva A. AI As a medical device: is it enough to ensure performance transparency and accountability in healthcare? SSRN. (2019) 1(1):5–16. 10.21552/eplr/2020/1/4

[55] Sartor G Lagioia F. The impact of the general data protection regulation (GDPR) on artificial intelligence (2020).

[56] Jamjoom AAB Jamjoom AMA Thomas JP Palmisciano P Kerr K Collins JW Autonomous surgical robotic systems and the liability dilemma. Front Surg. (2022) 9:1015367. 10.3389/fsurg.2022.1015367 36277285 PMC9580336

[57] Medtronic. UNID adaptive spine intelligence (ASI). Available at: https://www.medtronic.com/us-en/healthcare-professionals/products/spinal-orthopaedic/internal-fixation-systems/unid.html.

[58] Qiu K Zhao Z Haghiashtiani G Guo S-Z He M Su R 3D Printed organ models with physical properties of tissue and integrated sensors. Adv Mater Technol. (2018) 3(3):1700235. 10.1002/admt.201700235 29608202 PMC5877482

[59] Shieber J. Medivis has launched its augemented reality platform for surgical planning: TechCrunch (2019). Available at: https://tcrn.ch/2NbGMG5.

